# Improving access to services for psychotic patients: does implementing a waiting time target make a difference

**DOI:** 10.1007/s10198-020-01165-0

**Published:** 2020-02-25

**Authors:** Anika Kreutzberg, Rowena Jacobs

**Affiliations:** 1 Department of Health Care Management, Technical University of Berlin, Strasse des 17. Juni 135, 10623 Berlin, Germany; 2 Centre for Health Economics, University of York, Alcuin College, York, YO105DD UK

**Keywords:** Waiting time targets, Mental health, Early intervention in psychosis, Difference-in-difference analysis, C31, D04, I11

## Abstract

**Objective:**

In April 2015, the English National Health Service started implementing the first waiting time targets in mental health care. This study aims to investigate the effect of the 14-day waiting time target for early intervention in psychosis (EIP) services after the first six months of its implementation.

**Study design:**

We analyse a cohort of first-episode psychosis patients from the English administrative Mental Health and Learning Disabilities Dataset 2011 to 2015. We compare patients being treated by EIP services (treatment) with those receiving care from standard community mental health services (control). We combine non-parametric matching with a difference-in-difference approach to account for observed and unobserved group differences. We analyse the probability of waiting below target and look at different percentiles of the waiting time distribution.

**Results:**

EIP patients had an 11.6–18.4 percentage point higher chance of waiting below target post-policy compared to standard care patients. However, post-policy trends at different percentiles of the waiting time distribution were not different between groups.

**Conclusions:**

Mental health providers seem to respond to waiting time targets in a similar way as physical health providers. The increased proportion waiting below target did not, however, result in an overall improvement across the waiting time distribution.

**Electronic supplementary material:**

The online version of this article (10.1007/s10198-020-01165-0) contains supplementary material, which is available to authorized users.

## Introduction

Providing access to services for people in need of care is a key perspective for health systems around the world [1]. Hence, waiting times are of persistent policy concern in countries with National Health Service systems and universal access such as the United Kingdom, Canada, New Zealand, or Australia [2, 3]. Waiting lists can serve to stock available demand and optimise utilisation of the scarce supply of resources such as skilled staff and medical equipment [4]. However, excessively long waiting times risk poorer patient outcomes, create anxiety and disability during waiting [5–7] and threaten the desired principles of timely and equitable access to care [8].

A number of countries operate waiting time targets to guarantee patients access to care within a maximum window of time, even though the definition of this window varies widely across countries and areas of health care [1]. Since the National Health Service (NHS) Plan in 2000, the English NHS has had a sustained focus on continually setting shorter waiting time targets combined with aggressive performance management of providers. However, policy efforts so far have been limited to areas of physical health care such as elective surgery, and emergency care. In April 2015, the English Department of Health started implementing the first waiting time targets for selected mental health services [9]. First-episode psychosis patients were the first to be affected by a target. Fifty percent of patients being referred to an early intervention in psychosis (EIP) are guaranteed to wait no longer than 14 days from referral to treatment [10]. The EIP target was expected to be fully implemented by April 2016 and it is planned to be raised to 60% by 2020/21. A £40 million funding package was provided to support its implementation. Within the coming years, the intention is that all mental health services will be covered by a similar waiting time target.

This paper investigates the effects of the EIP target after the first six months of its implementation. We exploit the fact that patients with first-episode psychosis may receive care from two different service models: EIP care or standard community mental health care (standard care in the following). Whereas EIP patients are affected by the target policy, standard care patients are not and hence serve as our control group. Assuming that both groups would have common trends in the absence of the policy, the control group provides an estimate for the post-policy outcome of the treatment group had they not been affected by the target policy [11]. For the validation of the common trends assumption, comparability between groups is vital. To enhance comparability, we use control patients that are EIP-eligible but had no access to EIP services within 15 kms travel distance. We assume that a patient who is actually eligible for EIP care but would have to face a long travel distance to receive it would rather be treated by a comparable standard care service nearby but would not necessarily be different in terms of severity of the condition and need of treatment. To further ensure comparability between groups, we employ matching methods to control for observed characteristics [12] with a difference-in-difference regression model which further accounts for unobserved time-invariant components [13, 14]. We use coarsened exact matching [15] and propensity score matching [16] to show that results are robust against the choice of the matching method.

This is the first paper that evaluates the impact of a waiting time target in the mental health care context. Theoretically, the notion of provider responses to waiting time targets is motivated by a principal-agent economic model in the presence of asymmetric information. The policymaker (principal) wishes to maximise some welfare function that depends on an unobserved health outcome which can be influenced by the provider (agent)’s level of effort. Due to asymmetric information, the policymaker can only imperfectly observe the provider (agent)’s effort to achieve the unknown health outcome [17]. The waiting time target serves as a quantifiable measure to approximate the provider’s performance. Target performance is linked to some kind of financial or non-financial reward (or penalty) which incentivises the provider to achieve a good target performance. Empirical evidence has shown that providers do respond to waiting time targets in line with its intended objective [5, 6, 18]. Studies are, however, limited to state-level analyses in the area of physical health care. We contribute to the existing literature in a number of ways. First, our study moves beyond the state-level by analysing patient individual waiting times. This allows us to control for potential changes in case mix over time and further assures that both groups have been exposed to the same institutional setting. We analyse the probability of waiting below target at patient level and aggregate waiting times at provider level to analyse changes at different percentiles of the waiting time distribution. Data at provider level further allow us to test for some unintended provider responses to the target policies which have been investigated in the past [6, 19]. Therefore, we test whether providers decreased the length of treatment of existing patients or accepted fewer patients onto the caseload to free up resources and use them to improve target performance. Third, we choose a control group with no access to EIP services in a certain travel distance. For this, we create a novel dataset on the regional distribution of EIP and standard care services across England and calculate travel distances for patients. Third, we combine our difference-in-difference approach with non-parametric matching. Pre-processing the data through matching leads to less model dependence and reduced statistical bias in the regression analysis [20]. Finally, the EIP target operates in a different institutional setting to previous studies which may lead to different responses to performance targets. In contrast to single-event surgical procedures provided in hospitals, we provide evidence on services which are provided by stand-alone multidisciplinary teams within the community that deliver treatment in regular sessions over a period of up to 3 years [21]. Also, the need for treatment in the case of psychosis is acute rather than elective. Unlike target policies in the past, the EIP target is not accompanied with aggressive penalties but rather relies on the response of providers to the publication of performance data. Hence, we provide evidence on provider’s responses to performance targets without direct financial penalties [22, 23].

Our work will be of relevance to policymakers as it informs the future development of the English target policy and its potential international adaptation. We do not only provide novel information about EIP service availability and travel distances within the English NHS but also reveal and compare waiting times for both EIP and standard care patients for a large national cohort of first-episode psychosis patients. Hence, this study contributes to an ongoing debate as to whether specialised EIP services are superior to standard care in providing early access to care [24].

## Clinical and institutional background

Psychotic disorders are considered one of the most serious mental illnesses with tremendous economic and social consequences [25]. The first two to five years following the onset of psychosis are referred to as “first-episode psychosis” [26]. Here is where the majority of the decline in functioning emerges and treatment response is highest [27–29]. Treating first-episode psychosis requires a multidisciplinary approach including pharmacological, psychological, social, occupational and educational interventions [30]. Early intervention in psychosis (EIP) services have been developed to promote timely access to evidence-based care for first-episode psychosis patients [31, 32]. EIP services are stand-alone multidisciplinary teams within the community, specialised to promote early detection and treatment of first-episode psychosis and improve outcomes for affected patients. They are targeted at young people between the ages 16 and 35 to provide the best available treatments, support recovery and prevent relapse. Given the multidisciplinary nature, the care coordinator plays a key role in the effective delivery of EIP care [10, 33]. The care coordinator brings together all different professionals involved in the care of the patient and is responsible for engaging and supporting patients during treatment.

Although EIP services are well-established in many countries [31, 32], the universal availability of services is still lacking [33–36]. England has had a nationwide EIP implementation strategy from the early 2000s onwards [37]. However, service provision across the UK began to decline after continual financial constraints following initial funding. Insufficient funding for EIP care led to standard care services adopting methods from EIP services to improve treatment for patients with psychosis. At the same time, EIP services became less age-restrictive and merged their functions with standard care teams. Overall, the boundaries between both service models diluted over time [24]. EIP service provision varies geographically and significant numbers of young people across the country have no access to these services within a manageable travel distance. Travel distance is, however, important in this context as services are delivered with repeating service contacts over 2–5 years. Hence, EIP-eligible patients with no access to an EIP service close by end up using standard community mental health services (standard care) instead, to avoid travelling [38]. Standard care services are also stand-alone services within the community, offering mental health care in multidisciplinary teams. But unlike EIP services, they are not restricted to first-episode psychosis patients and accept patients of all ages. Both service models are provided by mental health trusts. There are just over 50 mental health care trusts within the English NHS (providers in the following). Each provider covers a certain geographical area with a number of inpatient wards as well as community-based service teams. That is, providers operate none or many EIP as well as standard care teams. The EIP waiting time target affects only patients being referred to EIP services which we exploit in our study design.

## Methods

### Difference-in-difference model

We use a difference-in-difference approach at the patient level to extract the effect of the EIP target on the probability of waiting below target (*Y*). For patient $i$ in provider $p$ at time $t$, we estimate the following model:

1 $$Y_{{{\text{ipt}}}} =\; \alpha + \beta {\text{POST}}_{t} + \theta {\text{TREAT}}_{{{\text{ip}}}} + \mu \left( {{\text{TREAT}}_{{{\text{ip}}}} \times {\text{POST}}_{t} } \right) + \gamma X_{{{\text{ipt}}}} + \sigma_{{\text{p}}} + \tau_{t} + \varepsilon_{{{\text{ipt}}}}$$

${\text{TREAT}}_{{{\text{ip}}}}$ is a dummy variable indicating whether the patient received EIP care, and $POST_{t}$ is a dummy variable for whether the patient was referred in the post-policy period. $X_{ipt}$ is a set of patient-level characteristics to account for time-varying differences in patient severity across the treatment and control groups and mitigate the effects of compositional changes over time. It contains the variables age, male, single, non-white, unemployed, no fixed accommodation, neighbourhood deprivation quintile (based on the Index of Multiple Deprivation [39], overall disease severity (based on the Health of the Nation Outcomes Scales (HoNOS) [40, 41]), severity of psychotic symptoms (HoNOS item 6), schizophrenia diagnosis, first-episode psychosis cluster, referral priority and referral source. A more detailed explanation of the variables can be found in Appendix 1. Fixed effects $\sigma_{{\text{p}}}$ for 58 mental health providers control for any time-invariant differences, and time fixed effects $\tau_{t}$ for 19 quarters account for any unobserved temporal fluctuations not related to the policy. $\varepsilon_{{{\text{ipt}}}}$ represents the idiosyncratic error.

The coefficient $\hat{\mu }$ yields the difference-in-difference estimate of the policy effect. It can be interpreted as the population average treatment effect which represents the expected gain from the target policy for an individual randomly selected from the treated population [14]. We expect the probability of EIP patients to wait below the target to increase in the post-policy period ($\hat{\mu } > 0$). We estimate Eq. (1) using a linear probability model and report robust standard errors that are clustered at the provider level.

In a second step, we aggregate our data at the provider level and analyse the policy effect at the mean, median, as well as at the 25th and 75th percentile of the waiting time distribution. We use ordinary least squares regression and included the same covariates as introduced above (aggregated at the mean within a provider). Further, we look at some potentially unintended effort substitution of providers due to the increased target pressure. Providers could, for example, decrease the length of treatment of existing patients or accept fewer patients onto the caseload to free up resources and use the additional resources to improve target performance [6]. Therefore, we analyse changes in the length of treatment and in the number of newly accepted patients onto the caseload using the same percentiles and including the same covariates as introduced above.

### Pre-processing the data through matching

The credibility of the difference-in-difference approach in identifying the policy effect depends on the comparability of the treatment and control group in terms of observed as well as unobserved characteristics. In our case, the assignment to EIP and standard care is not random. Patients access services through various routes [42]. Most commonly they will be referred by a health professional. A small proportion may also have self-referred. Whereas EIP services are exclusive to first-episode psychosis patients between the ages of 16 and 35, standard care is not limited to psychotic conditions and patients may enter services at all ages. Hence, we expect patients in the treatment group to be younger and have a more severe or further developed psychotic condition than standard care patients.

We use matching as a non-parametric method to balance the treatment and control groups in terms of potentially confounding pre-treatment control variables before applying our regression model. We perform two different well-established matching methods: coarsened exact matching (CEM) and propensity score matching (PSM). CEM matches a treated unit to all the control units with the same covariate values to ensure common support over the covariates [15]. However, the more covariates there are to be matched, the less likely it is to find a suitable control unit. As a consequence, unmatched treatment units have to be excluded from the analysis and the estimated treatment effect is redefined to the area of common support [14]. In contrast, PSM is an approximate matching method that identifies control units which are close to the treated unit in terms of the propensity score, i.e. the probability of being treated conditional on the covariates [43]. This less restrictive method allows for more treatment units to remain in the final estimation sample. We conduct a one-to-one propensity score matching with replacement and enforcing common support.

In both approaches, we match on patient demographic factors (age, male, single, non-white, neighbourhood deprivation quintile) as well as on variables related to the patient’s psychotic condition (severity of psychotic symptoms, schizophrenia diagnosis, first-episode psychosis cluster). Matched units were assigned a weight which was entered as an inverse probability weight to the regression based on Eq. (1). Any residual difference in the groups after matching was accounted for by the patient characteristics vector in the model. We assessed balance by* t* tests of mean differences for individual covariates, and the reduction in standardized percentage bias [16].

### Validation of the difference-in-difference approach

The difference-in-difference method assumes common time trends for both the treated and the control group [14]. This means that in the absence of treatment, the average change in the outcomes would be the same for treated as for untreated individuals. If the assumption is violated, the estimated treatment effect would be confounded with a natural time trend. We examine the assumption by testing whether linear pre-policy trends are statistically different between the treatment and the control group. If both groups have common trends prior to the policy, then there is a reasonable expectation that outcomes would also change post-policy at similar rates in the absence of the intervention [11, 44]. Hence, we re-run the regression based on Eq. (1) including a full set of quarter dummies and an interaction of the dummies with the treatment indicator to model differential trends for treatment and control groups.

The assumption would further be violated if waiting times already changed prior to the policy implementation, in anticipation of the policy change. In October 2014, EIP services were officially announced for the first time to be affected by a target. To deal with potential anticipatory bias we, therefore, omit the two quarters from October 2014 to the start of implementation in April 2015 from the analysis.

Another requirement for our difference-in-difference approach to be valid is that the comparison group is not affected by the intervention. That is, the target policy does not spill-over from EIP services to standard care services [44]. Since mental health providers may offer both, EIP and standard care, there is a possibility of spill-over effects in two directions. First, providers may re-allocate resources to enhance EIP target performance at the expense of poorer standard care performance. Second, the increased effort to improve access for EIP patients will lead to improvements in access for standard care patients as well. To investigate the possibility of any spill-over effects we make use of the fact that some providers in our sample offer standard care only. Whereas providers offering both service models and thus experiencing target pressure for their EIP patients may spill-over resources, providers offering standard care only are less likely to be affected by the EIP target policy. Hence, we repeat our main analysis with a control group that is limited to patients being with providers that only offer standard care, to see whether we observe the same policy effect as for the full sample.

Additionally, we compare standard care outcomes pre- and post-policy for providers that offer both service types (treatment) with those that offer standard care only (control). The model is identical to Eq. (1) with the only difference being the treatment indicator. We use the same matching procedure, outcome variables and estimation methods as introduced above.

## Data and measures

### Sample

We use secondary data from the administrative Mental Health and Learning Disabilities Dataset (MHLDDS). The MHLDDS contains patient-level data on any mental health-related treatment in hospitals and community settings within the English NHS [45].^1^ Since 2003, data collection is mandatory for all providers of specialist mental health services funded by the English NHS. For the purpose of this analysis, we have access to data from April 2011 to the end of data collection in November 2015.

We define the pre-policy period from April 2011 to September 2014 (14 quarters), and post-policy from April 2015 to November 2015 (3 quarters). The period of anticipation from October 2014 to March 2015 was omitted. In accordance with the policy guideline, our treatment group includes patients aged 16–35 years and being referred to an EIP service [10]. Standard care patients are identified by having had a community mental health care episode within the study period. To select EIP-eligible patients from this group, we combined a number of criteria which have been used in previous literature [38, 46]. Standard care patients must have had either a diagnosis of schizophrenia, been classified into the first-episode psychosis cluster or reported problems associated with hallucinations and delusions. Further, we limit our control group to EIP-eligible patients that had no access to EIP services within 15 kms travel distance. We assume that a patient who is actually eligible for EIP care but would have to face a long travel distance to receive it would rather be treated by a comparable standard care service nearby. This patient would, however, not necessarily be different from an EIP patient in terms of severity of condition and need for treatment.

The MHLDDS provides the Health of the Nation Outcomes Scales (HoNOS) as a routinely collected outcome measure for severe mental health conditions [40, 41]. HoNOS has been found to be significantly associated with waiting times for first-episode psychosis patients [47]. The measure consists of 12 items of which item six indicates the severity of problems with hallucinations and delusions. The score ranges from 0 (no problems) to 4 (very severe problems). Since the measure was important to ensure comparability between groups in terms of symptom severity, we exclude patients with missing HoNOS records from the analysis.

### Outcome measures

The policy guideline monitors the time from referral to treatment [10]. Treatment is defined as the patient’s acceptance onto the caseload and the assignment of a care coordinator. Thus, we measure referral-to-treatment waiting time as the days from referral to care coordinator assignment. Referrals within the MHLDDS cannot be directly linked to the service they were directed to. We used a number of measures to identify the referral directed to the relevant EIP episode. The MHLDDS defines care spells which are overarching and continuous periods of time a patient spent in the care of a single or multiple healthcare providers [48]. We considered all care spells that started within the study period and where the patient’s first team episode was with an EIP service. We identified referrals that initiated the care spell (i.e. happened before the start of the spell). Referrals could have been received from multiple sources, including primary and secondary care providers, other tertiary mental health or social care providers, agencies within the justice system and self-referrals. We considered only referrals that were accepted by the receiving provider. If there were multiple accepted referrals before the start of a care spell, we used the referral closest to the start of the care spell. We used the first care coordinator the patient was assigned to following the start of an EIP episode to stop the waiting time clock. Based on the estimated referral-to-treatment waiting time, we created a dummy that equals 1 if the waiting time was 14 days or less, and 0 otherwise. Length of treatment was measured as the number of days from start to end of the first EIP or standard care episode (recurrent episodes not included). We use the logarithm of waiting time and length of treatment to account for the right-sided skewness.

### Service availability and travel distances

The MHLDDS provides information on the mental health provider the patient was receiving care from and the type of care (EIP or standard care). However, no information is available on how many EIP and standard care teams a provider has and which of the teams the patient received care from. To identify providers that offer both or only one of the service models as well as to calculate travel distances for patients, we generated a novel dataset on the number and location of EIP and standard care teams per provider across England. We manually researched all provider websites to collect address information of all relevant service teams and double-checked whether the identified teams were registered as a site with an NHS (or care) provider based on information published online by NHS Digital. Based on this list, we calculated travel distances from the patient’s place of residence to the nearest EIP team (which is not necessarily the one a patient was receiving care from). We measured distance in a straight line from the geographical centroids of the 2001 LSOA to the grid reference of the service’s postcode using Stata 14 MATA.

## Results

### Descriptive statistics

In total, we identified 17,472 EIP and 23,554 EIP-eligible standard care patients. We included 5625 (32%) EIP patients with valid HoNOS records. From the 12,404 (53%) standard care patients with a valid HoNOS record, we selected 3702 (30%) that had no access to EIP care. In Appendix 2 and 3, we compare characteristics of the included and excluded patients. Patients excluded with missing HoNOS had a longer waiting time but also showed fewer other indicators of a psychosis such as a schizophrenia diagnosis or a first-episode psychosis cluster episode which may indicate that these patients are not clearly patients with psychosis and are better excluded. Standard care patients with access to EIP (excluded) were more likely to live in the most deprived neighbourhoods.

Table 1 compares the sample characteristics of both groups before and after matching. Before matching, *t* tests indicate the groups to be highly imbalanced on all observed characteristics. The EIP group was on average three years younger and more likely to be male, single, non-white, and from more deprived neighbourhoods. EIP patients also had more severe problems with hallucinations and delusions (HoNOS six score) and were more likely to be diagnosed with schizophrenia or allocated to the first-episode psychosis care cluster. Although some differences in group means remain after matching, the observed mean bias between the two groups reduced substantially from 39.1 to 17.1 after CEM and 4.9 after PSM, respectively. PSM seems to have performed better particularly in balancing the psychosis related characteristics.

**Table 1:** Sample characteristics before and after matching

Patient characteristic	Unmatched		Matched controls	
	Treated	Controls	CEM	PSM
Age (mean)	22.7	26.0***	22.4*	22.5*
Male (%)	0.66	0.48***	0.66	0.64
Single (%)	0.95	0.89***	0.98***	0.96
Non-white ethnicity (%)	0.32	0.20***	0.19***	0.33
Least deprived quintile (%)	0.11	0.17***	0.13**	0.14***
Second least deprived quintile (%)	0.14	0.19***	0.14	0.14
Third least deprived quintile (%)	0.18	0.23***	0.17	0.20**
Fourth least deprived quintile (%)	0.23	0.22	0.23	0.20***
Most deprived quintile (%)	0.34	0.19***	0.32	0.32
HoNOS 6 score (range 0–4, mean)	1.99	1.51***	1.66***	1.78***
Schizophrenia diagnosis (%)	0.20	0.06***	0.03***	0.18
First-episode psychosis cluster (%)	0.72	0.11***	0.47***	0.72

*CEM *Coarsened exact matching, *PSM *Propensity score matching

*p < 0.05, **p < 0.01, ***p < 0.001 for *p* values of *t* tests of mean differences between groups

Table 2 summarises the proportion below target and mean waiting times by treatment status. Independent of the matching approach, EIP patients had a significantly higher chance of waiting below target during the whole study period. Also, mean waiting times are considerably shorter for EIP patients compared to EIP-eligible standard care patients.

**Table 2:** Proportion below target and mean waiting times by treatment status

	Proportion below target		Waiting time in days	
	Treated	Control	Treated	Control
Unmatched	0.289	0.209***	48.6	81.7***
Coarsened exact matching	0.289	0.202***	48.6	106.8***
Propensity score matching	0.289	0.205***	48.1	105.0***

*p < 0.05, **p < 0.01, ***p < 0.001 for *p* values of t-tests of mean differences between groups

Table 3 shows that mean waiting time for patients below the target increased post-policy in both groups. At the same time, mean waiting times for patients above the target improved considerably post-policy.

**Table 3:** Mean waiting time and standard deviations in days conditional on being below the target

	Pre-policy period		Post-policy period	
	Mean	SD	Mean	SD
All EIP patients	52.0	113.1	22.8	26.4
EIP patients below target	5.6	4.5	7.1	4.6
EIP patients above target	83.8	138.1	37.2	29.8
All standard care patients	84.9	173.5	30.1	33.3
Standard care patients below target	4.9	4.3	6.2	4.2
Standard care patients above target	133.5	205.4	46.1	34.7

**Fig. 1:**
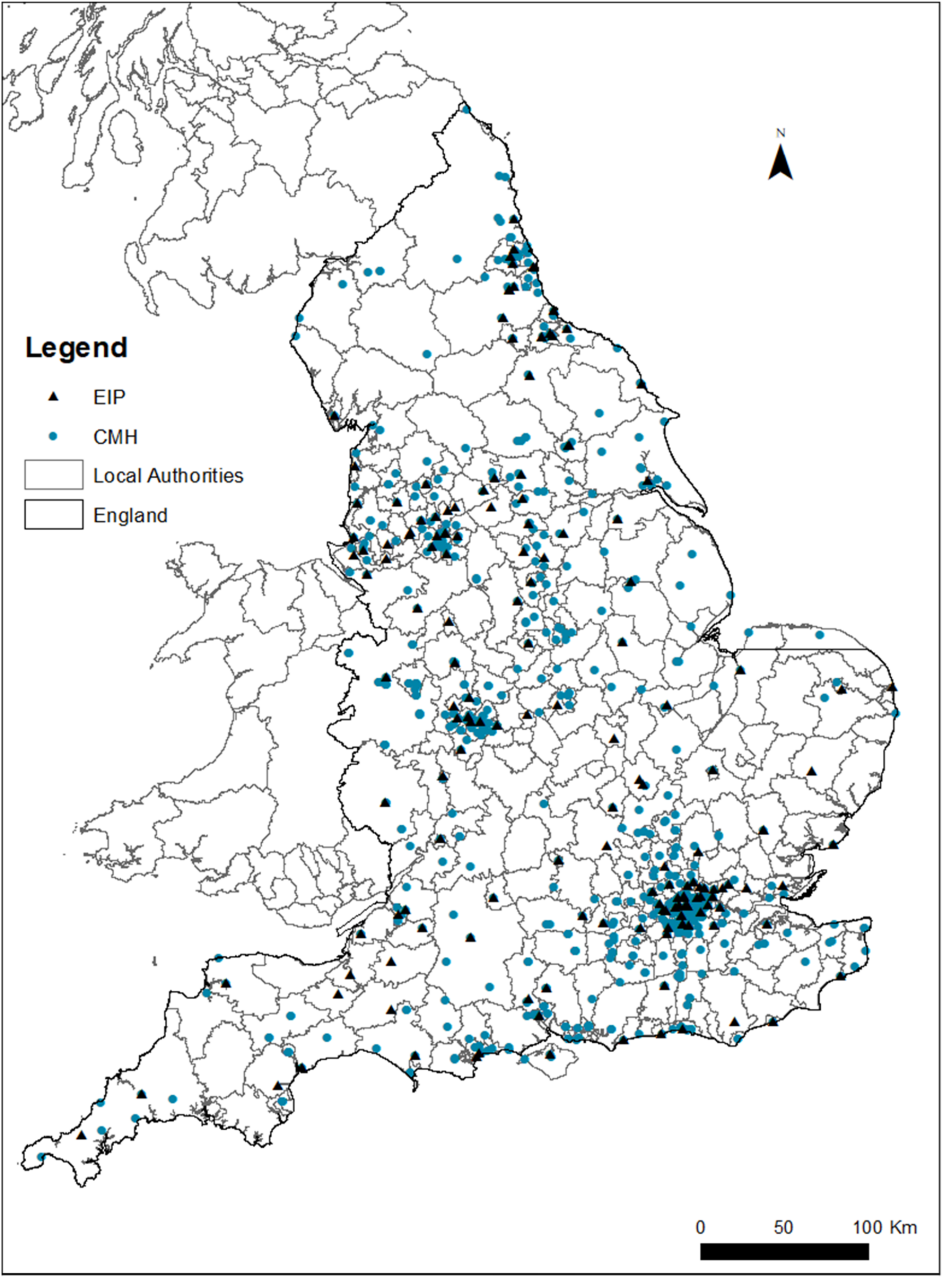
Regional distribution of EIP and standard care (CMH for community mental health) service availability in England

There are 58 providers in the sample with an average of 3 EIP teams and 13 standard care teams. 13 providers offered standard care only. Appendix 4 compares the patient case mix of providers offering both care types with those offering standard care only, before matching. Patients of providers offering standard care only were more likely female, married, of White ethnicity and from least deprived neighbourhoods. They also had less severe problems with hallucinations and delusions (HoNOS 6). Figure 1 maps the distribution of EIP and standard care (CMH for community mental health) services across England. The average travel distance of EIP patients to their nearest EIP service was 11 kms with a minimum of 0.9 and a maximum of 87 kms. 50% lived no more than 7 kms, 75% no more than 15 kms, and 90% no more than 25 kms away from the nearest EIP service. Travel distance to the nearest EIP service is shorter for patients in most deprived neighbourhoods (8 kms) compared to 12–13 kms for EIP patients from the least deprived neighbourhoods.

**Fig. 2:**
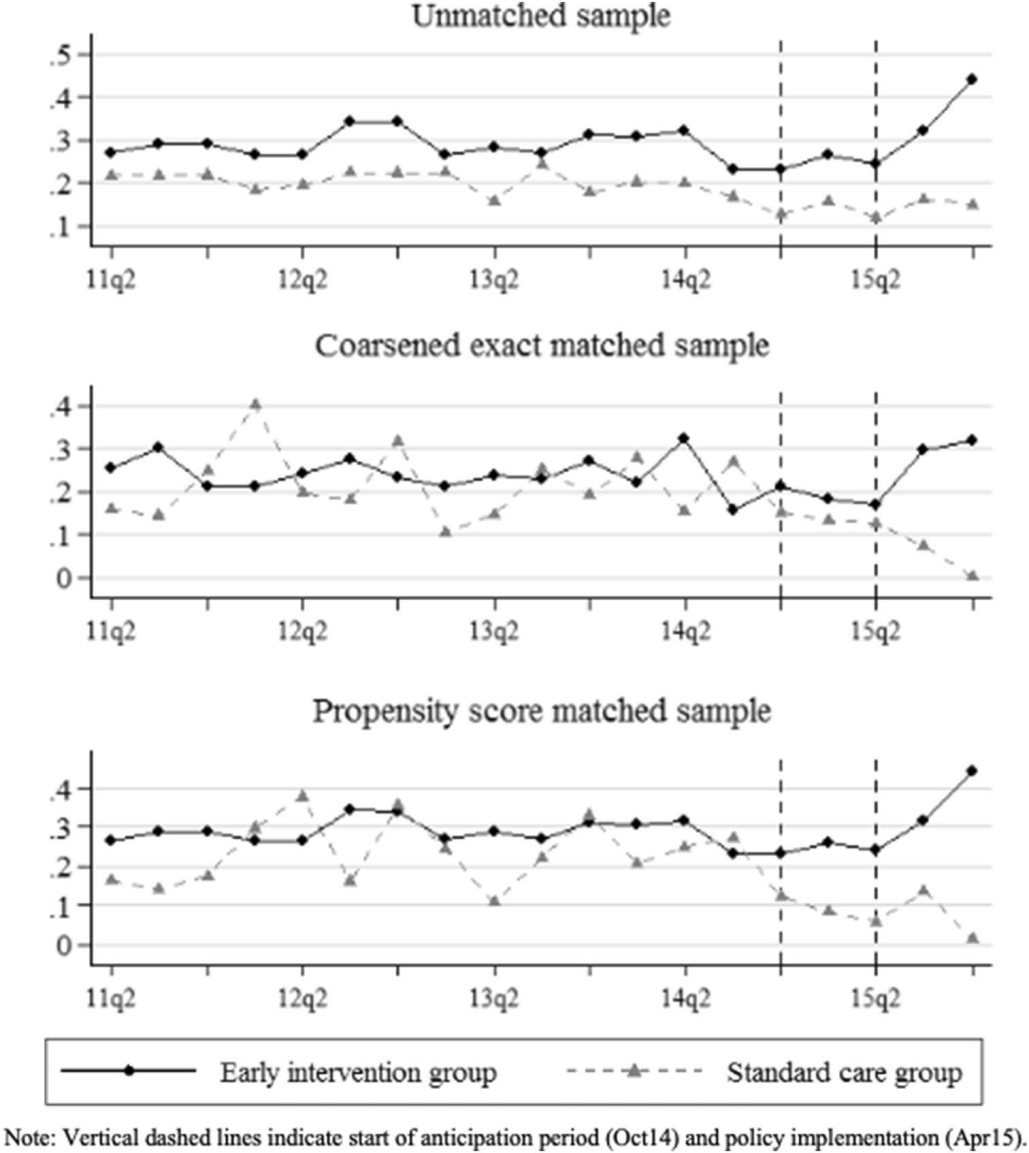
Pre- and post-policy trends by treatment group before and after matching

Figure 2 visualises pre- and post-policy trends of the probability of waiting below target for EIP and standard care patients before and after matching. Trends are quite stable and parallel between the groups between 2011 and 2013. We observe a slight downward trend in outcomes for both groups starting around the second quarter of 2014. Whereas this downward trend continued for the control group post-policy, the probability of waiting below target increased for EIP patients after the policy implementation.

**Fig. 3:**
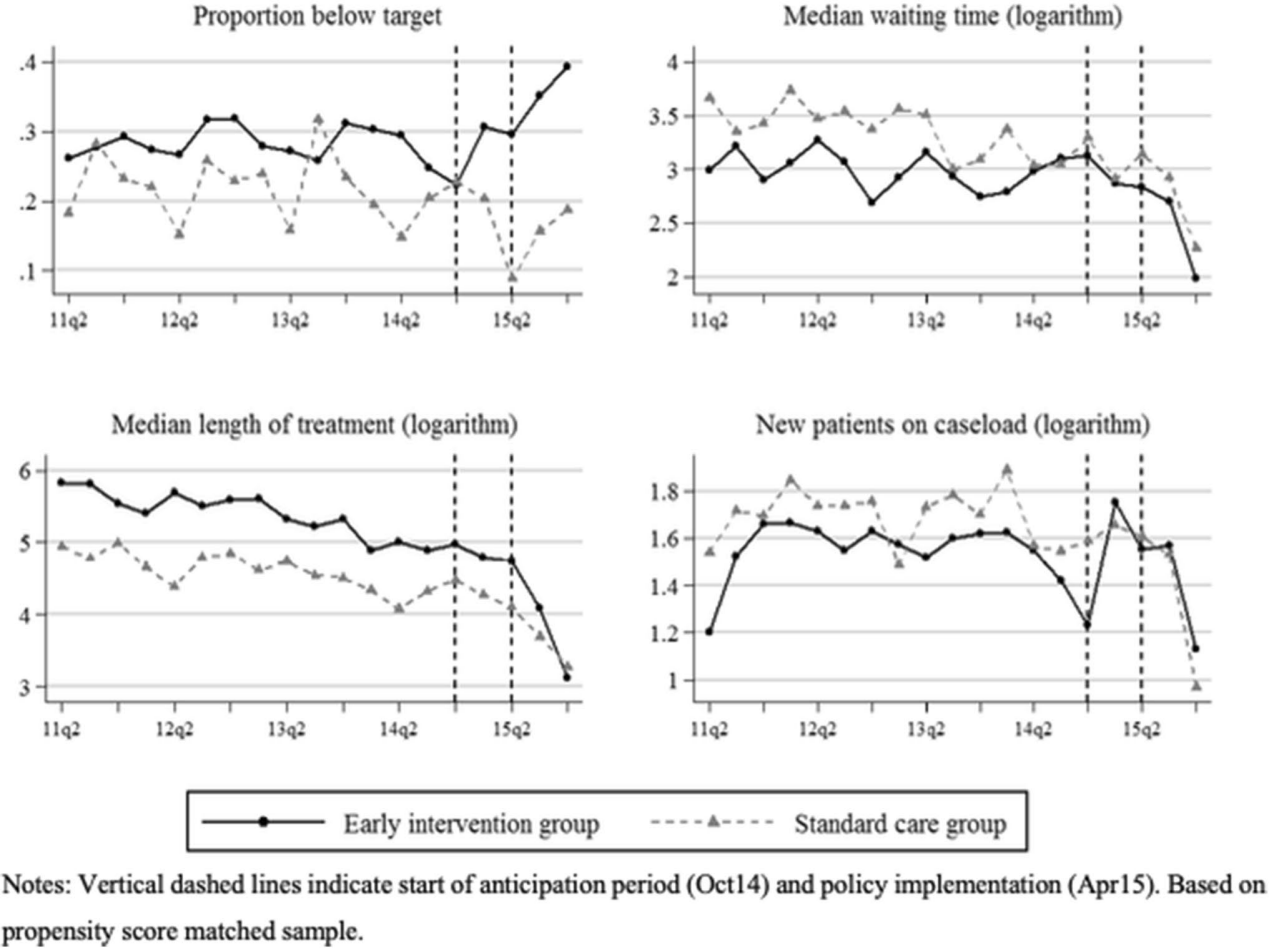
Provider-level pre- and post-policy trends in outcomes by treatment group

In Fig. 3, we present pre- and post-policy trends of outcomes aggregated at the provider level (based on the propensity score-matched sample). We observe a similar downward trend in the proportion of patients waiting below target shortly before the start of the anticipation period and a strong increase post-policy for both groups as in the patient-level case. Again, the EIP group exceeded its pre-policy levels whereas the standard care group recovered to their pre-policy levels before the downward trend. For median waiting time (logarithm) and median length of treatment (logarithm), we see a constant downward pre-policy trend for both groups which continued during the period of anticipation and increased post-policy. There is no clearly identifiable trend in pre-policy numbers of new patients accepted onto the caseload for both groups. It appears that numbers dropped slightly after the anticipation of the policy change.

### Estimation results

Table 4 reports the patient-level estimation results from Eq. (1). We find a significant positive post-policy effect for EIP patients on the probability of waiting below target independent of the matching method. EIP patients had an 11.6–18.4 percentage point higher chance of waiting below target post-policy compared to standard care patients. Full regression results can be found in Appendix 5.

**Table 4:** Patient-level difference-in-difference results of the EIP target policy effect on the probability to wait below target

	(1) Unmatched sample		(2) Coarsened exact matching		(3) Propensity score matching	
Post-policy	0.064	(0.058)	− 0.076	(0.082)	0.014	(0.060)
EIP patient	0.019	(0.040)	0.032	(0.043)	0.015	(0.049)
Post-policy for EIP	0.116*	(0.049)	0.168**	(0.061)	0.184**	(0.068)
Observations	8393		3712		6873	

*p < 0.05, **p < 0.01, ***p < 0.001. Regression based on Eq. (1). Pre-policy: Apr11 to Mar15; post-policy: Apr15-Nov15. Oct14-Mar15 omitted. Cluster robust standard errors in parentheses

We observe a similarly consistent effect on the proportion of waiting below target at the provider-level, independent of the matching method (see Table 5, panel 1). The proportion of EIP patients waiting below target increased by 13.8–16.5 percentage points per provider post-policy. However, there was no policy effect on the median waiting time (panel 2). Estimates show that median waiting times were significantly lower for EIP patients compared to standard care patients and decreased post-policy. But this decrease appears to have been similarly strong for both groups. We also could not find any policy effect for other parts of the waiting time distribution such as the 25th and 75th percentile or the mean (results not reported).

**Table 5:** Provider-level difference-in-difference results of the EIP target policy effect on various outcomes

	(1) Unmatched sample		(2) Coarsened exact matching		(3) Propensity score matching	
(1) Proportion below target						
Post-policy	− 0.028	(0.107)	0.036	(0.032)	0.057	(0.044)
EIP patient	− 0.010	(0.047)	0.020	(0.065)	0.043	(0.063)
Post-policy for EIP	0.157**	(0.053)	0.165**	(0.056)	0.138*	(0.059)
Observations	1527		1400		1468	
(2) Median waiting time (logarithm)						
Post-policy	− 0.313	(0.595)	− 0.486**	(0.162)	− 0.411	(0.205)
EIP patient	− 1.284***	(0.232)	− 1.421***	(0.296)	− 1.260***	(0.288)
Post-policy for EIP	− 0.071	(0.198)	0.083	(0.253)	0.049	(0.245)
Observations	1392		1,214		1,303	
(3) Median length of treatment (logarithm)						
Post-policy	0.929	(0.587)	0.452*	(0.183)	0.674	(0.221)
EIP patient	− 2.213***	(0.178)	− 1.961***	(0.210)	− 2.048***	(0.185)
Post-policy for EIP	− 0.377*	(0.150)	− 0.313	(0.159)	− 0.447**	(0.159)
Observations	1527		1400		1468	
(4) New patients on caseload (logarithm)						
Post-policy	− 1.089***	(0.368)	− 0.576***	(0.145)	− 0.286	(0.152)
EIP patient	− 0.577**	(0.185)	− 0.523**	(0.151)	− 0.516**	(0.155)
Post-policy for EIP	0.282	(0.149)	0.240	(0.141)	0.248	(0.130)
Observations	1527		1400		1468	

*p < 0.05, **p < 0.01, ***p < 0.001. Regression based on Eq. (1). Pre-policy: Apr11 to Mar15; post-policy: Apr15-Nov15. Oct14 to Mar15 omitted. Cluster robust standard errors in parentheses

We find some evidence that the EIP target caused a decrease in the median length of treatment for EIP patients (panel 3). This decrease already started pre-policy but continued to be significantly different for EIP patients compared to standard care patients post-policy. We observe the same effect for the 75th but not for the 25th percentile of the distribution (results not reported). In contrast, we find no evidence that EIP providers accepted fewer patients onto their caseloads than standard care providers post-policy (panel 4).

### Validation checks

A limitation of using the linear probability model to estimate our difference-in-difference equation is that the predicted values may be outside the [0,1] interval. Appendix 6 shows that only a small proportion of predicted values (0.04–0.09) lie below zero and one value lies above one.

The analysis of pre-policy trends showed no significant difference between the two comparison groups. Appendix 7 presents the treatment of specific referral quarter estimates for both the CEM and the PSM matched samples. From the non-significant pre-policy trends, we conclude that the common trends assumption is likely to hold. We do, however, observe significantly different trends during the time of anticipation which confirms our approach to exclude the quarters of anticipation from the main analysis.

Results from the test of potential spill-overs from EIP to standard care services are presented in Appendix 8. We observe the same positive policy effect on the probability of waiting below target when limiting the control group to patients that were with providers offering standard care only and after using coarsened exact matching (panel 1; coefficient: 0.200; *p* < 0.05). The significance of the effect disappears once we use the PSM sample. It needs to be noted, that the number of controls is very small due to the additional exclusion criterion (683/776 controls after CEM/PSM). Comparing standard care outcomes of providers offering both service models to those offering standard care only did not show any significant differences in post-policy trends (see Appendix 8, panel 2). Overall, we conclude that the impact of any spill-over effects if any was small.

## Discussion

Access to specialist services at the early stages of psychosis is critical to successful treatment and recovery. EIP services are internationally recognised as supporting the timely provision of evidence-based care to patients with psychosis. However, in times of financial constraints EIP services may not always be able to meet the desired standards of providing rapid access for patients in need. To tackle increasing waiting times, the English government pioneered the introduction of a waiting time target for EIP services. This paper examines the effectiveness of this target policy in improving access for first-episode psychosis patients. We made use of a difference-in-difference design which is a well-established method to evaluate the impact of health policy interventions in the absence of randomized controlled trial data. We found the EIP target to be effective in increasing the number of patients waiting below target in the first six months of its implementation. However, waiting times across the whole distribution have not changed differently compared to standard care patients.

We find some evidence of the reduced length of treatment for EIP patients. This may be explained by the fact that the shorter waiting time allowed patients to recover faster. But it could also be the result of an unintended effort substitution due to the increased target pressure. Providers may have referred patients earlier to follow-up mental health care teams or transferred very severe cases earlier into hospitals for inpatient treatment to free up resources and treat waiting patients faster. At the same time, we find no evidence that providers accepted fewer patients onto their caseload to meet targets.

Our research moves beyond previous work on the effectiveness of waiting time targets which is limited to country-level comparisons as we are able to compare two patient groups being treated within the same institutional setting. This allows us to measure and compare waiting times at the patient level and thus adequately control for changes in case mix over time and between groups. The challenge lies in ensuring comparability between the groups in terms of variables that may also be associated with waiting time. We select control patients with no access to EIP services within a certain travel distance to improve the comparability of groups. Furthermore, the combination of matching and the difference-in-difference design allows us to balance the groups observed as well as unobserved confounders. Whereas the regression model accounted for any remaining imbalances after matching through adding additional covariates, the non-parametric matching helped to reduce model dependence and statistical bias. We found our matching approach to reduce bias in observed characteristics between the two groups substantially. Validation checks further indicated that the common trends assumption was likely to hold and potential spill-overs between EIP and standard care patients was negligible.

There are some limitations to our research. First, our post-policy period is relatively short due to the fact that the collection for MHLDDS temporarily stopped in November 2018 to introduce a revised dataset version from April 2019 onwards which was not yet available for research at the time of this analysis. Hence, we are only able to look at the first six months of the implementation process. Over time, effects may either become larger once more providers respond to the target policy at later stages, or effects may disappear over time as providers only temporarily focus on the newly introduced target. However, the target policy was announced months before its implementation so most providers will have prepared for the change to hit the target early on. Also, this research provides immediate evidence for policymakers to guide future development of the target policy. As more service areas are expected to be affected by similar waiting time targets in future, the interplay between the responses to the different targets by a provider will challenge future research in this area. Second, our estimation of waiting time is imperfect. Where patients had a number of referrals, we chose the referral closest to the start of the care spell. This may have underestimated waiting time if earlier referrals were relevant to the psychotic episode. If EIP patients had systematically more referrals than standard care patients (or vice versa) this could have biased our results. Thus, our results have to be interpreted under the assumption that this possible measurement error is randomly distributed across the two patient groups. To understand the different referral pathways of patients until they receive EIP care would be a fruitful follow-up research question. Third, despite various measures to improve comparability between the groups, our results may still be driven by group differences that we were not able to account for but had an impact on patient waiting times. By matching the two groups by small-area deprivation we aimed to reduce any potential bias as far as possible. However, the exclusion of standard care patients with access to EIP services within 15 kms may have systematically excluded control patients from more urban areas where access to health care, in general, is better (and thus waiting times are shorter). But EIP services are not necessarily concentrated in urban areas. On the contrary, the EIP service framework aims to provide access to care within the community and hence where health care provision, in general, is low. In practice, EIP provision is mainly driven by financial support from the responsible Clinical Commissioning Group and is quite opaque. Our attempt to draw a map of EIP services across England is the first of its kind and the information gathered deserves further exploration in future research. Our measured treatment effect is defined for patients under common support—so for patients that were comparable in terms of observed characteristics. We cannot conclude from our results to what extent the estimated effect is generalizable to the whole population of EIP patients. The decision to exclude patients with no observed HoNOS information may have further limited the generalizability of our results. Particularly, if severity was related to deprivation and deprivation was related to access to care (and thus waiting times) results may have been biased. However, the sample selection criteria and the procedure to exclude patients with missing HoNOS information is commonly used in literature working with the same dataset and patient group [38]. Overall, using the national administrative database allowed us to draw our estimation sample from a nationally representative patient cohort including a large number of mental health providers across England. This is an advantage compared to the existing literature in the area of psychosis which usually relies on much smaller, regionally limited patient cohorts from only one or two providers.

The routine collection of referral-to-treatment waiting times in the new MHLDDS release from April 2019 (called MHSDS) is a positive development towards more comprehensive research in this area. It will allow a more accurate measurement of waiting time, a better identification of relevant patients and the exploration of policy effects over time. A more comprehensive study would further benefit from other control groups. Previous literature, for example, used Scotland—with no targets in place—as a comparison at country level [5, 6]. But this requires other comparable countries to collect EIP waiting times. The EIP target is planned to be tightened by 2020/21 and other mental health services shall be affected by similar targets in the future. For research purposes, it would be desirable to implement these anticipated policy changes stepwise in clearly defined regions (or trusts) so that a comparison of EIP patients between targeted and non-targeted regions is possible. Also, our observed changes in the length of treatment as a potential strategy of providers to substitute effort being under target pressure deserves some further analysis.

Our research will be of great relevance to policymakers not only in England but internationally. Psychosis is associated with a high degree of disability, anxiety and discomfort. Further, psychosis has a significant social and economic dimension as people suffering from acute psychotic phases have difficulties in fulfilling their family and work commitments [25, 49]. Intervening early has shown to improve outcome prospects in various dimensions and corresponding EIP services have emerged worldwide [29, 32]. However, many countries report insufficient access to EIP services nowadays [34–36]. England has a history of success in operating waiting time targets and significantly reducing waiting times in the physical health sector. Our work provides an important starting point to find out whether this success can be translated into the mental health sector. Mental illness, given its impact on individuals, health services, the economy and society, is growing in importance for policymakers. At the same time, waiting times for mental health-related treatment are a growing concern for many countries. We show that targets can be an effective means to improve access to specialised mental health care. But at the same time, unintended effects on outcomes for existing patients need to be monitored. Our research can help inform the future development of the EIP target and its expansion to other areas of mental health in England, as well as informing policymakers in other countries considering the introduction of a similar policy.

## Electronic supplementary material

Below is the link to the electronic supplementary material.

Supplementary file1 (DOCX 65 kb)

## Footnotes

1 A previous version was called Mental Health Minimum Dataset. From September 2014, the dataset was renamed Mental Health and Learning Disabilities Dataset following the inclusion of people in contact with learning disability services. Data collection paused in November 2015 in order to introduce a new version, the Mental Health Services Dataset, from April 2016.
