# Supplementary material for: Improving access to services for psychotic patients: does implementing a waiting time target make a difference
Source: Eur J Health Econ. 2020 Feb 25;21(5):703–16. doi: 10.1007/s10198-020-01165-0 (PMC7366592; doi:10.1007/s10198-020-01165-0)
Supplement: Supplementary file 1 — Supplementary file1 (DOCX 65 kb) [file 10198_2020_1165_MOESM1_ESM.docx]

**Appendix 1** Definition of variables used in the regression models

| Variable | Definition |
| --- | --- |
| Post-policy | Equals 1 if referral happened in the post-policy period (from April 2015) and 0 otherwise |
| EIP patient | Equals 1 if patient received EIP care and 0 if patient received standard community care |
| Post-policy for EIP | Represents the interaction between the post-policy and the EIP patient indicators |
| Age | Age at referral, continuous variable, restricted to age 16 to 35 |
| Single | Equals 1 if marital status is single, separated or divorced and 0 if other marital status |
| Male | Equals 1 if gender is male and 0 if other gender |
| Non-white ethnicity | Equals 1 if ethnicity is any non-white ethnicity and 0 if white ethnicity |
| No fixed accommodation | Equals 1 if no fixed accommodation and 0 if fixed accommodation |
| Unemployed | Equals 1 if no employment and 0 if employed, student or house carer |
| Neighbourhood deprivation quintiles | Categorical variable representing the 5 neighbourhood deprivation quintiles (from 1=least deprived to 5=most deprived) based on the Index of Multiple Deprivation 2010 which captures deprivation at lower super output area (LSOA) level. Reference category is the least deprived quintile. |
| Schizophrenia diagnosis | Equals 1 if patient received a schizophrenia diagnosis and 0 if any other mental illness diagnosis or diagnosis unknown |
| First-episode psychosis cluster | Equals 1 if the patient was categorised into the first-episode psychosis cluster and 0 if otherwise. Mental healthcare clusters are reference groups used to group service users with similar needs and problem severities related to their mental health within the English NHS. |
| Overall disease severity | Measured by the Health of the Nation Outcomes Scales (HoNOS) which is a routinely collected, clinician rated measure of mental health severity. The score is composed of 12 items. It ranges from 0 (no problems) to 48 (most severe problems). |
| Psychotic symptom severity | Item 6 of HoNOS measures problems with hallucinations and delusions which we use as a measure of psychotic symptom severity. It ranges from 0 (no problems) to 4 (very severe problems). |
| Interaction between overall and psychotic disease severity | An interaction term between the total HoNOS score and the HoNOS item 6. |
| Referral source | Categorical variable representing whether the referral source was general practitioner or other primary care (most common and reference category), self-referral, internal referral (same trust), other NHS mental health trust, acute secondary care, other agency (e.g. justice system), or unknown referral source. |

**Appendix 2** Comparison of patients with HoNOS score (included) and without HoNOS score (excluded)

|  | Treated |  | Controls |  |
| --- | --- | --- | --- | --- |
| Outcome variable | HoNOS | No HONOS | HoNOS | No HONOS |
| Proportion below target | 0.26 | 0.18*** | 0.18 | 0.17 |
| Waiting time in days | 50.5 | 78.9*** | 82.9 | 110.3*** |
| Patient characteristic |  |  |  |  |
| HoNOS 6 score (mean) | - | - | - | - |
| Schizophrenia diagnosis (%) | 0.17 | 0.12*** | 0.09 | 0.08 |
| First-episode psychosis cluster (%) | 0.66 | 0.45*** | 0.13 | 0.12* |
| Age (mean) | 22.2 | 21.9*** | 26.3 | 26.2 |
| Male (%) | 0.62 | 0.64** | 0.47 | 0.51*** |
| Single (%) | 0.95 | 0.97*** | 0.88 | 0.91*** |
| Non-white ethnicity (%) | 0.36 | 0.33* | 0.31 | 0.30* |
| Least deprived quintile (%) | 0.12 | 0.11* | 0.13 | 0.11* |
| Second least deprived quintile (%) | 0.14 | 0.14 | 0.14 | 0.13 |
| Third least deprived quintile (%) | 0.17 | 0.18 | 0.19 | 0.18 |
| Fourth least deprived quintile (%) | 0.23 | 0.23 | 0.26 | 0.25 |
| Most deprived quintile (%) | 0.34 | 0.35 | 0.28 | 0.32*** |
| Note: * p<0.05, ** p<0.01, *** p<0.001 for p-values of t-tests of mean differences between groups. | | | | |

**Appendix 3** Comparison of standard care patients with no access to EIP (included) and with access to EIP (excluded)

| Outcome variable | No access | Access |
| --- | --- | --- |
| Proportion below target | 0.21 | 0.19** |
| Waiting time in days | 81.7 | 75.4 |
| Patient characteristic |  |  |
| HoNOS 6 score (mean) | 1.51 | 1.52 |
| Schizophrenia diagnosis (%) | 0.06 | 0.08 |
| First-episode psychosis cluster (%) | 0.11 | 0.11** |
| Age (mean) | 26.0 | 26.6*** |
| Male (%) | 0.48 | 0.50* |
| Single (%) | 0.89 | 0.90 |
| Non-white ethnicity (%) | 0.20 | 0.33*** |
| Least deprived quintile (%) | 0.17 | 0.10*** |
| Second least deprived quintile (%) | 0.19 | 0.12*** |
| Third least deprived quintile (%) | 0.23 | 0.17*** |
| Fourth least deprived quintile (%) | 0.22 | 0.27*** |
| Most deprived quintile (%) | 0.19 | 0.34*** |
| Note: * p<0.05, ** p<0.01, *** p<0.001 for p-values of t-tests of mean differences between groups. | | |

**Appendix 4** Patient case mix of providers offering both care types and providers offering standard care only

|  | Provider offers | |
| --- | --- | --- |
| Patient characteristic | EIP care and standard care | Standard care only |
| Age (mean) | 25.3 | 25.2 |
| Male (%) | 0.55 | 0.52* |
| Single (%) | 0.92 | 0.90* |
| Non-white ethnicity (%) | 0.31 | 0.23*** |
| Least deprived quintile (%) | 0.12 | 0.15*** |
| Second least deprived quintile (%) | 0.14 | 0.15 |
| Third least deprived quintile (%) | 0.19 | 0.20 |
| Fourth least deprived quintile (%) | 0.25 | 0.25 |
| Most deprived quintile (%) | 0.31 | 0.26*** |
| HoNOS 6 score (mean) | 1.68 | 1.52*** |
| Schizophrenia diagnosis (%) | 0.11 | 0.09** |
| First-episode psychosis cluster (%) | 0.31 | 0.27** |

**Appendix 5** Full regression results of the patient-level difference-in-difference estimation on the probability to wait below target

|  | (1) Unmatched sample | | (2) Coarsened Exact Matching | | | (3) Propensity Score Matching | | | |
| --- | --- | --- | --- | --- | --- | --- | --- | --- | --- |
| Post-policy | 0.064 | (0.058) | -0.076 | (0.082) | | 0.014 | | (0.060) | |
| EIP patient | 0.019 | (0.040) | 0.032 | (0.043) | | 0.015 | | (0.049) | |
| Post-policy for EIP | 0.116* | (0.049) | 0.168** | (0.061) | | 0.184** | | (0.068) | |
|  |  |  |  |  | |  | |  | |
| Demographics |  |  |  |  | |  | |  | |
| Age | 0.001 | (0.001) | -0.001 | (0.002) | | -0.001 | | (0.002) | |
| Single | -0.005 | (0.016) | 0.002 | (0.039) | | 0.000 | | (0.051) | |
| Male | 0.011 | (0.008) | 0.026 | (0.015) | | -0.004 | | (0.020) | |
| Non-white ethnicity | -0.001 | (0.008) | 0.005 | (0.021) | | 0.015 | | (0.022) | |
| No fixed accommodation | 0.019 | (0.016) | 0.019 | (0.037) | | 0.035 | | (0.030) | |
| Unemployed | 0.002 | (0.011) | 0.010 | (0.026) | | -0.002 | | (0.029) | |
|  |  |  |  |  | |  | |  | |
| Neighbourhood deprivation quintiles | (reference category: least deprived deprivation quintile) | | | | | |  | | |
| Second least deprived quintile | -0.012 | (0.017) | -0.009 | (0.033) | | -0.011 | | (0.025) | |
| Third least deprived quintile | -0.019 | (0.017) | 0.000 | (0.039) | | 0.025 | | (0.037) | |
| Fourth least deprived quintile | -0.008 | (0.016) | 0.014 | (0.034) | | 0.054 | | (0.030) | |
| Most deprived quintile | -0.016 | (0.018) | -0.009 | (0.033) | | 0.014 | | (0.031) | |
|  |  |  |  |  | |  | |  | |
| Psychosis related variables |  |  |  |  | |  | |  | |
| Schizophrenia diagnosis | 0.042* | (0.018) | -0.039 | (0.061) | | 0.019 | | (0.035) | |
| First-episode psychosis cluster | 0.092** | (0.017) | 0.071** | (0.021) | | 0.081 | | (0.022) | |
| Total HoNOS score | 0.001 | (0.002) | 0.003 | (0.002) | | 0.000 | | (0.003) | |
| HoNOS 6 score | 0.017 | (0.010) | 0.028 | (0.017) | | -0.001 | | (0.024) | |
| Interaction between HoNOS and HoNOS 6 | 0.000 | (0.001) | -0.002 | (0.001) | | 0.000 | | (0.001) | |
|  |  |  |  |  | |  | |  | |
| Referral source | (reference category: general practitioner or other primary care) | | | | | |  | | |
| Self-referral | -0.013 | (0.043) | -0.023 | (0.054) | | -0.021 | | (0.039) | |
| Internal referral (same trust) | 0.054 | (0.026) | 0.127** | (0.041) | | 0.094 | | (0.042) | |
| Other NHS mental health trust | -0.008 | (0.079) | 0.004 | (0.090) | | -0.020 | | (0.090) | |
| Acute secondary care | -0.047 | (0.026) | -0.019 | (0.051) | | -0.074 | | (0.032) | |
| Other agency | -0.042 | (0.032) | -0.063 | (0.061) | | -0.094 | | (0.038) | |
| Referral source not known | -0.027 | (0.020) | -0.019 | (0.022) | | -0.010 | | (0.022) | |
|  |  |  |  |  | |  | |  | |
| *Coefficients for 58 provider dummies and 19 referral quarter dummies are not reported here.* | | | | |  | |  | |  |
|  |  |  |  |  | |  | |  | |
| Observations | 8,393 |  | 3,712 |  | | 6,873 | |  | |
| Note: * p<0.05, ** p<0.01, *** p<0.001. Regression based on equation (1). Pre-policy: Apr11 to Mar15; post-policy: Apr15-Nov15. Oct14-Mar15 omitted. Cluster robust standard errors in parentheses. | | | | | | | | | |

**Appendix 6** Observed and predicted probabilities of waiting below target

|  | n | Mean | Std. dev. | Min. | Max. | n (%) if | n (%) if |
| --- | --- | --- | --- | --- | --- | --- | --- |
|  |  |  |  |  |  | fitted <0 | fitted >1 |
| **Unmatched sample** |  |  |  |  |  |  |  |
| observed probability | 8,393 | 0.263 | 0.440 | 0 | 1 |  |  |
| fitted probability | 8,393 | 0.263 | 0.202 | -0.145 | 0.972 | 364 (0.04) | 0 (0.00) |
| **Coarsened exact matched** |  |  |  |  |  |  |  |
| observed probability | 3,712 | 0.227 | 0.419 | 0 | 1 |  |  |
| fitted probability | 3,712 | 0.218 | 0.205 | -0.214 | 1.024 | 339 (0.09) | 1 (0.00) |
| **Propensity score matched** |  |  |  |  |  |  |  |
| observed probability | 6,873 | 0.269 | 0.444 | 0 | 1 |  |  |
| fitted probability | 6,873 | 0.261 | 0.211 | -0.203 | 0.951 | 480 (0.07) | 0 (0.00) |

**Appendix 7** Referral quarter estimates from the test of common trends for proportion below target

|  | (1) Coarsened exact matching | | (2) Propensity score matching | | |
| --- | --- | --- | --- | --- | --- |
| Pre-policy |  |  |  |  | |
| 11q3 | 0.028 | (0.070) | 0.089 | (0.109) | |
| 11q4 | 0.078 | (0.064) | 0.024 | (0.055) | |
| 12q1 | 0.042 | (0.043) | 0.124 | (0.065) | |
| 12q2 | -0.003 | (0.045) | 0.095 | (0.052) | |
| 12q3 | 0.018 | (0.042) | 0.053 | (0.063) | |
| 12q4 | 0.042 | (0.046) | 0.096 | (0.065) | |
| 13q1 | 0.070 | (0.057) | 0.087 | (0.063) | |
| 13q2 | -0.047 | (0.044) | 0.071 | (0.073) | |
| 13q3 | 0.057 | (0.067) | 0.134 | (0.083) | |
| 13q4 | 0.057 | (0.062) | 0.110 | (0.068) | |
| 14q1 | 0.093 | (0.071) | 0.101 | (0.068) | |
| 14q2 | -0.048 | (0.058) | 0.064 | (0.070) | |
| 14q3 | -0.066 | (0.054) | -0.026 | (0.047) | |
| Anticipation |  |  |  |  | |
| 14q4 | -0.108 | (0.055) | -0.014 | (0.051) | |
| 15q1 | -0.042 | (0.042) | 0.022 | (0.054) | |
| Post-policy |  |  |  |  | |
| 15q2 | -0.076 | (0.041) | -0.087 | (0.048) | |
| 15q3 | -0.03 | (0.056) | 0.047 | (0.054) | |
| 15q4 | 0.018 | (0.083) | 0.115 | (0.088) | |
| Pre-policy for EIP |  |  |  |  | |
| 11q2 for EIP | 0.052 | (0.041) | 0.122 | (0.060) | |
| 11q3 for EIP | 0.057 | (0.073) | 0.048 | (0.089) | |
| 11q4 for EIP | -0.014 | (0.066) | 0.089 | (0.070) | |
| 12q1 for EIP | 0.005 | (0.044) | 0.004 | (0.055) | |
| 12q2 for EIP | 0.067 | (0.051) | 0.020 | (0.057) | |
| 12q3 for EIP | 0.072 | (0.052) | 0.110 | (0.061) | |
| 12q4 for EIP | 0.055 | (0.060) | 0.075 | (0.071) | |
| 13q1 for EIP | -0.011 | (0.068) | 0.035 | (0.068) | |
| 13q2 for EIP | 0.097 | (0.063) | 0.055 | (0.071) | |
| 13q3 for EIP | 0.008 | (0.066) | -0.007 | (0.067) | |
| 13q4 for EIP | 0.059 | (0.060) | 0.045 | (0.056) | |
| 14q1 for EIP | -0.039 | (0.073) | 0.042 | (0.065) | |
| 14q2 for EIP | 0.157* | (0.061) | 0.108 | (0.067) | |
| 14q3 for EIP | 0.106 | (0.063) | 0.120* | (0.052) | |
| Anticipation for EIP |  |  |  |  | |
| 14q4 for EIP | 0.153* | (0.063) | 0.121* | (0.050) | |
| 15q1 for EIP | 0.118* | (0.045) | 0.121** | (0.042) | |
| Post-policy for EIP |  |  |  |  | |
| 15q2 for EIP | 0.140** | (0.051) | 0.221*** | (0.050) | |
| 15q3 for EIP | 0.193*** | (0.067) | 0.157** | (0.055) | |
| 15q4 for EIP | 0.209* | (0.093) | 0.204* | (0.082) | |
| Note: * p<0.05, ** p<0.01, *** p<0.001. "11q3" equals quarter 3 in year 2011. Reference category is 11q2. Models includes all covariates based on Equation (1). Cluster robust standard errors in parentheses. | | | | |  |

**Appendix 8** Test of spill-overs from EIP to standard care services

| (1) Treatment = EIP patient | (a) Coarsened exact matching | | (b) Propensity score matching | |
| --- | --- | --- | --- | --- |
| Post-policy | -0.066 | (0.101) | -0.098 | (0.128) |
| EIP patient | 0.073 | (0.129) | 0.238 | (0.138) |
| Post-policy for EIP | 0.200* | (0.087) | 0.299 | (0.154) |
| Observations | 1,879 |  | 5,797 |  |
| (2) Treatment = Provider that offers EIP and standard care | (a) Coarsened exact matching | | (b) Propensity score matching | |
| Post-policy | -0.039 | (0.068) | -0.058 | (0.040) |
| Treatment | 0.119*** | (0.013) | 0.055* | (0.022) |
| Post-policy for treatment | -0.045 | (0.054) | 0.046 | (0.063) |
| Observations | 4,533 |  | 10,480 |  |
| Note: * p<0.05, ** p<0.01, *** p<0.001. Pre-policy: Apr11 to Mar15; post-policy: Apr15-Nov15. Oct14-Mar15 omitted. Based on Equation (1) but: in model (1) control group limited to providers that offer standard care only; in model (2) treatment =1 if patients were with providers that offer EIP services additionally to standard care. Cluster robust standard errors in parentheses. | | | | |
